# Advances in interleukin-10-based therapies for pulmonary diseases: focus on targeted lung delivery systems

**DOI:** 10.3389/fimmu.2025.1630990

**Published:** 2025-11-12

**Authors:** Weikun Tian, Xu Wang, Jia Zeng, Ya Gao, Shuo Tang, Caifeng Ma, Liping Ye, Xinghan Tian

**Affiliations:** 1 School of Clinical Medicine, Shandong Second Medical University, Weifang, China; 2 The Affiliated Yantai Yuhuangding Hospital of Qingdao University, Yantai, China; 3 Second Clinical Medical College of Binzhou Medical University, Yantai, China; 4 Yantai Yuhuangding Hospital, Yantai, China

**Keywords:** interleukin-10, pulmonary inflammation, anti-inflammatory, targeted delivery, nanoparticles, hydrogels, extracellular vesicles, cell-based delivery

## Abstract

Interleukin-10 (IL-10) is an anti-inflammatory cytokine that exerts diverse effects on immune regulation. It alleviates excessive inflammatory responses in the body by inhibiting the expression of pro-inflammatory cytokines and the activation of antigen-presenting cells. In recent years, the therapeutic potential of IL-10 in various pulmonary inflammatory diseases has attracted extensive attention, including acute lung injury (ALI), acute respiratory distress syndrome (ARDS), asthma, and pulmonary fibrosis. IL-10 has also been studied in lung transplantation to improve the pro-inflammatory cytokine profile of donor lungs that do not meet conventional criteria. Nonetheless, its limited bioavailability, short half-life and potential for systemic adverse effects constrain its clinical application. To enhance its therapeutic efficacy and lung tissue targeting, intranasal administration and nebulized inhalation are the earliest methods applied in pulmonary diseases. Recombinant proteins, engineered mesenchymal stem cells, nanoparticle delivery systems, and gel delivery systems have also been developed and are undergoing preclinical trials. Many drug delivery platforms and pulmonary-targeted approaches have been shown to effectively increase the drug’s accumulation in the lungs and sustain its release, thus minimizing systemic toxicity. These IL-10-based therapies for pulmonary diseases can be broadly categorized into two main strategies: prolonging the half-life of exogenous IL-10 and enhancing the secretion of endogenous IL-10. The former mainly includes the development of IL-10 fusion proteins, nanoparticle delivery systems, and hydrogel delivery systems. The latter primarily involves IL-10 expression plasmids and IL-10-expressing adenoviruses. Despite its therapeutic potential, the clinical translation of IL-10 remains challenging. Its narrow therapeutic window constrains efficacy, and factors such as patient heterogeneity, disease stage, and the dynamic regulation of IL-10 signaling complicate the establishment of optimal dosing regimens. Emerging targeted delivery strategies provide opportunities to overcome these limitations by enabling precise spatial and temporal modulation of IL-10 activity. In light of these opportunities and challenges, this review aims to provide a comprehensive overview of current IL-10 delivery systems and to highlight strategies for their optimization to facilitate clinical translation in pulmonary diseases.

## 1 Introduction

Inflammation-associated lung diseases are pulmonary conditions in which inflammation plays a central role in the pathogenesis, progression, or exacerbation of the disease. These diseases often involve immune cell infiltration, cytokine production, tissue remodeling, and impaired gas exchange (1). Pneumonia remains a major global cause of illness and mortality, with particularly high death rates among children under five and adults over seventy. In 2021, excluding COVID-19, the global mortality rate from lower respiratory tract infections across all age groups was 27.7 per 100,000 population, resulting in a total of 2.18 million deaths (2). ALI is a critical condition characterized by neutrophil infiltration in the lung tissue, damage to the alveolar-capillary barrier, and pulmonary edema. These manifestations can lead to severe arterial hypoxemia and impaired carbon dioxide clearance (3). ARDS, a critical form of ALI, is associated with hospital mortality rates exceeding 40%, with major contributors including persistent pulmonary inflammation, disruption of the vascular endothelial barrier, continuous alveolar edema, and the progression to multiple organ dysfunction (4). The annual incidence of ARDS ranges from 3.65 to 78.9 cases per 100,000 population. The mortality rate of non-COVID-related ARDS remains relatively stable, at approximately 30%–35% for mild ARDS and 45%–50% for severe ARDS (5). Chronic obstructive pulmonary disease (COPD) is commonly associated with an increase in B cell numbers, specifically in the small airway lymphoid follicles (6). It is a chronic respiratory disease characterized by persistent airflow obstruction and respiratory symptoms, and it is the third leading cause of death worldwide. As of 2017, the number of people with chronic respiratory diseases worldwide reached 544.9 million, of which approximately 55% of cases were attributed to COPD (7). Asthma is a chronic respiratory inflammation caused by intermittent bronchospasm, leading to dyspnea and wheezing, affecting approximately 300 million people globally. As of 2023, the prevalence of asthma is approximately 10% among children and adolescents worldwide, and around 6%–7% among adults (8). Idiopathic pulmonary fibrosis (IPF) is the leading type of interstitial lung disease. IPF typically leads to respiratory failure, with a median survival of only 2.5 to 3.5 years (9). A major challenge in the treatment of IPF is diagnostic delay, with the typical time from symptom onset to IPF diagnosis being 1.7 years (10). The abnormal deposition of collagen and other extracellular matrix (ECM) components within the lung parenchyma is a pathological hallmark of idiopathic pulmonary fibrosis (IPF). Transforming growth factor-β (TGF-β), released during epithelial cell injury, and its downstream mediator connective tissue growth factor (CTGF), contribute to the increased ECM accumulation, which often leads to impaired gas exchange and altered lung function (11).

IL-10 is a multifunctional anti-inflammatory cytokine that plays a crucial role in inhibiting excessive immune responses and maintaining immune homeostasis (12). However, the therapeutic use of IL-10 encounters multiple obstacles in clinical settings, such as its short half-life *in vivo* (13), pro-inflammatory side effects due to its pleiotropic effects (14), and the need for precise delivery to the site of pulmonary inflammation. To overcome these limitations, researchers have developed various IL-10 therapeutic strategies, including IL-10 gene therapy, fusion proteins, and nanoparticle delivery systems.

**Figure 1:**
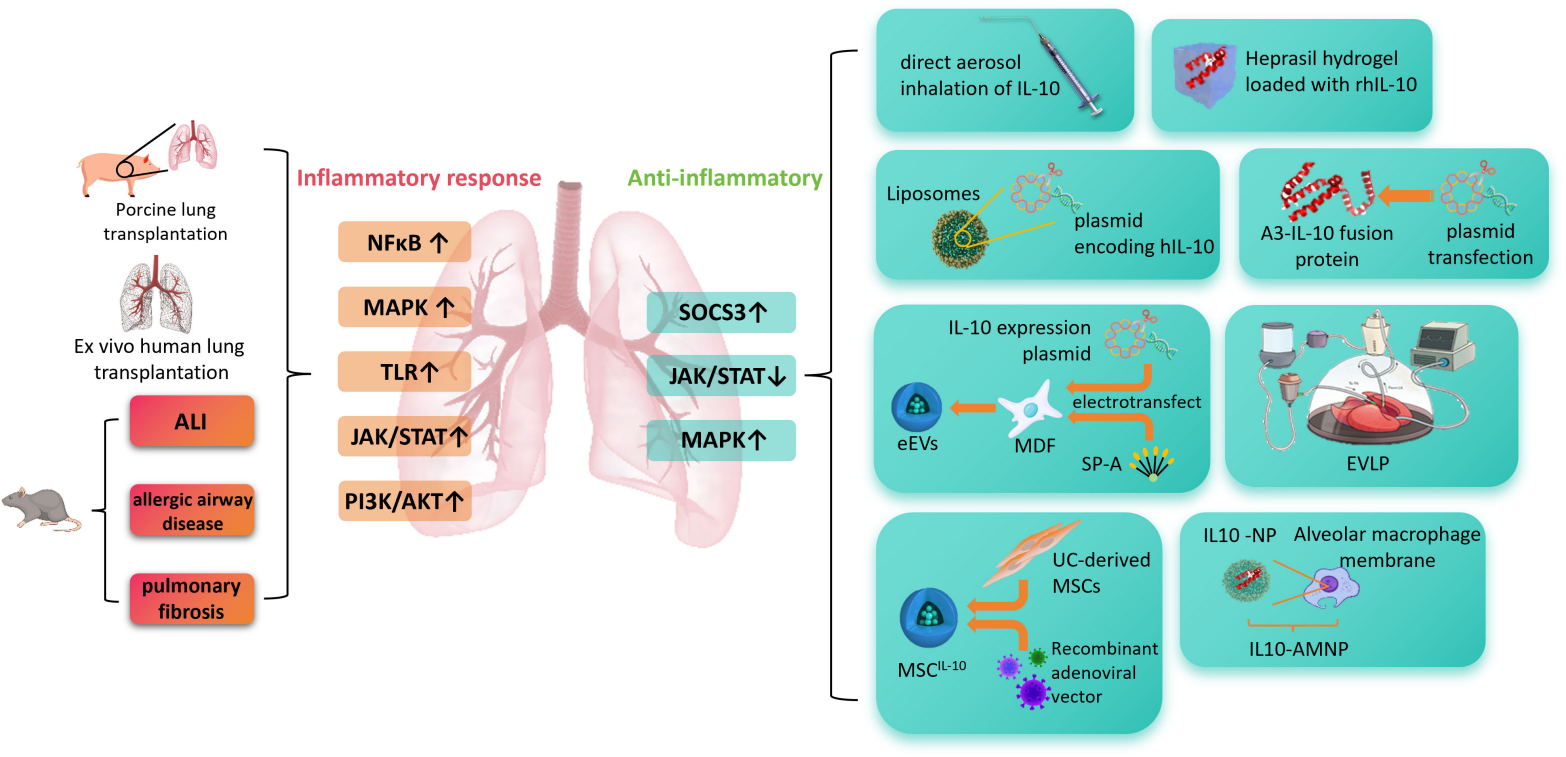
This figure illustrates the role of IL-10 in pulmonary inflammation-related diseases and its delivery strategies. In pathological conditions such as acute lung injury, allergic airway disease, and pulmonary fibrosis, signaling pathways including NF-κB, MAPK, TLR, JAK/STAT, and PI3K/AKT are excessively activated, leading to inflammatory responses. IL-10 exerts anti-inflammatory effects by activating SOCS3 and modulating JAK/STAT and MAPK pathways. Various IL-10 delivery approaches are shown on the right, including aerosol inhalation, hydrogel-based release, liposome/plasmid delivery, A3-IL-10 fusion protein, electroporation with ex vivo lung perfusion, mesenchymal stem cell (MSC)-based vectors, and nanoparticle systems combined with macrophage membranes, which enhance its stability and targeting efficiency. ALI, acute lung injury; NF-κB, nuclear factor kappa-light-chain-enhancer of activated B cells; MAPK, mitogen-activated protein kinase; TLR, Toll-like receptor; JAK/STAT, Janus kinase/signal transducer and activator of transcription; PI3K/AKT, phosphoinositide 3-kinase/protein kinase B; SOCS3, suppressor of cytokine signaling 3; rIL-10, recombinant interleukin-10; hIL-10, human interleukin-10; A3-IL-10, von Willebrand factor A3 domain–interleukin-10 fusion protein; MDF, mouse dermal fibroblasts; SP-A, surfactant protein A; eEVs, engineered extracellular vesicles; EVLP, ex vivo lung perfusion; UC-MSCs, umbilical cord-derived mesenchymal stem cells; MSC^IL-10^, mesenchymal stem cells expressing interleukin-10; NP, nanoparticle; IL10-AMNP, interleukin-10–alveolar macrophage membrane nanoparticle.

This article provides a concise overview of the immunomodulatory functions of IL-10 and its therapeutic potential in a variety of lung diseases. We discuss current IL-10-based therapeutic modalities and emphasize recent advancements in lung-targeted delivery strategies, with the goal of offering theoretical and technical insights to support the further development of IL-10 as an effective treatment for lung inflammation-associated disorders. **Figure 1** summarizes the core arguments of this review in a graphical abstract.

## 2 Therapeutic effects of IL-10

Since the identification of the IL-10 family, researchers have been working to design IL-10-based therapeutic approaches for a range of conditions, including autoimmune disorders, infections, tissue damage and cancer (15). IL-10 is widely expressed in various cells from both the innate and adaptive immune systems. Macrophages, monocytes, dendritic cells (DCs), natural killer (NK) cells, CD4+ and CD8+ T cells, as well as B cells, all express IL-10 (16). IL-10 is the first cytokine identified in the major three subfamilies of the IL-10 family (17) and is initially named cytokine synthesis inhibitory factor (CSIF). It is secreted by activated CD4+ T helper (Th) 2 cells and is known to inhibit the synthesis of Th1 cytokines (18).

### 2.1 Regulation of immune inflammation by IL-10

IL-10 and its receptors are expressed in vertebrates, forming an ancient anti-infection mechanism that constitutes the inflammation resolution phase of host defense (19). IL-10 primarily targets antigen-presenting cells (APCs) such as monocytes and macrophages, inhibiting their release of pro-inflammatory cytokines and chemokines. This mainly includes tumor necrosis factor-α (TNF-α), IL-1β, granulocyte colony-stimulating factor (G-CSF), granulocyte-macrophage colony-stimulating factor (GM-CSF), MCP-1, and IP-10 (20). IL-10’s inhibitory effects on IL-1 and TNF are crucial for its anti-inflammatory activity, as these cytokines synergize in inflammation and amplify the inflammatory response by inducing secondary mediators such as chemokines, prostaglandins, and platelet-activating factor (PAF, 16). IL-10 interferes with antigen presentation primarily by reducing the expression of major histocompatibility complex II (MHC II), co-stimulatory molecules, and adhesion molecules (21, 22). When directly acting on T cells, IL-10 can induce their anergy, suppressing T cell proliferation and cytokine production (23). IL-10 can also inhibit IL-12 and IL-23, which are required for CD4+ T cell differentiation (24).

**Figure 2:**
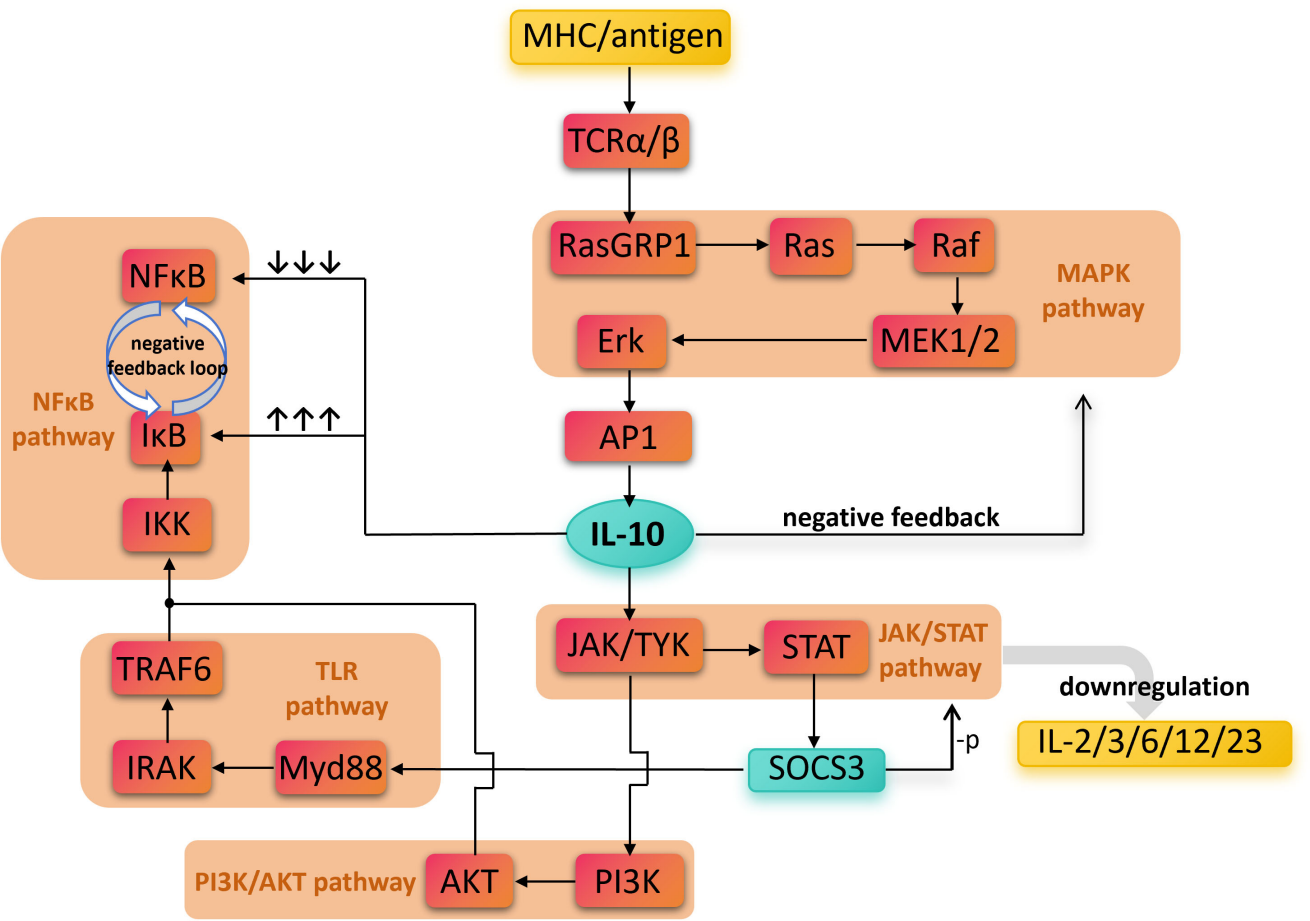
The direct and indirect biochemical pathways involved in IL-10’ s anti-inflammatory effects. Antigen recognition activates multiple pro-inflammatory pathways, including TCR, NF-κB, TLR, and PI3K/AKT, leading to inflammatory responses. In contrast, IL-10 signals through the JAK/STAT pathway to induce SOCS3 expression, which suppresses the production of pro-inflammatory cytokines such as IL-2, IL-3, IL-6, IL-12, and IL-23. Overall, the figure emphasizes IL-10 as a key anti-inflammatory mediator that negatively regulates inflammatory signaling, maintains immune homeostasis, and prevents excessive immune damage. Nuclear Factor kappa-light-chain-enhancer of activated B cells, NF-κB; Mitogen-Activated Protein Kinase, MAPK; Toll-Like Receptor, TLR; Phosphoinositide 3-Kinase/Protein Kinase B, PI3K/AKT; Janus Kinase/Signal Transducer and Activator of Transcription, JAK/STAT; Major Histocompatibility Complex, MHC; T Cell Receptor alpha/beta, TCRα/β; Ras Guanyl Releasing Protein 1, RasGRP1; Rat sarcoma viral oncogene homolog, Ras; Rapidly Accelerated Fibrosarcoma kinase, Raf; Mitogen-Activated Protein Kinase Kinase 1/2, MEK1/2; Extracellular Signal-Regulated Kinase, Erk; Activator Protein 1, AP-1; IκB Kinase, IKK; Inhibitor of κB, IκB; TNF Receptor-Associated Factor 6, TRAF6; Interleukin-1 Receptor-Associated Kinase, IRAK; Myeloid Differentiation Primary Response 88, Myd88; Phosphoinositide 3-Kinase, PI3K; Protein Kinase B (also called PKB), AKT; Janus Kinase/Tyrosine Kinase, JAK/TYK; Signal Transducer and Activator of Transcription, STAT; Suppressor of Cytokine Signaling 3, SOCS3; Interleukin, IL.

**Figure 2** provides a simplified illustration of the downstream pathways involving IL-10 and its anti-inflammatory mechanisms.

### 2.2 Therapeutic potential of IL-10 in pulmonary diseases

In a study using a mouse ALI model, Huan Qin and colleagues (25) demonstrated that direct inhalation of rhIL-10 effectively ameliorated pulmonary cytokine storm. Interventions using placenta-derived mesenchymal stem cells (pMSCs, 26), human fetal lung-derived mesenchymal stem cells (hFL-MSCs, 27), or carnitine palmitoyltransferase 1 (CPT1A, 28) have all found that the suppression of the ALI inflammatory response is IL-10-mediated, and blocking the IL-10 signaling pathway exacerbates the inflammatory response in ALI (29). ARDS is the most severe form of ALI, often caused by pulmonary infections, trauma, inflammation, or hemorrhagic shock (30). IL-10 also shows strong therapeutic potential in ARDS caused by COVID-19 (31).

COPD is a classic example of chronic pulmonary inflammation. The reduced numbers of IL-10-secreting regulatory B cells (IL-10^+^ B-reg, 32) and IL-10-secreting regulatory T cells (IL-10^+^ T-reg, 33) in COPD patients indicate that IL-10 expression is negatively correlated with the progression of COPD. In a rat model of COPD, the alleviation of pulmonary inflammation induced by allopurinol was mediated through the enhancement of endogenous IL-10 (34). In a cell-based study simulating chronic airway inflammation, fish oil intervention reduced cellular oxidative stress by enhancing the expression of IL-10 (35). These findings suggest that IL-10 could serve as a biomarker for assessing the severity of COPD. The exact mechanism of IL-10 in human asthma remains unclear, but a decrease in IL-10 expression has been closely linked to the exacerbation of asthma in clinical patients, as well as increased eosinophils and circulating IgE levels (36). A study on viral infections leading to childhood asthma also confirmed the correlation between decreased IL-10 levels and asthma incidence (37). Insufficient IL-10 production by T cells is considered a contributing factor to allergic asthma, as it impairs the regulation of the Th2 response (38). Current clinical antifibrotic treatments are unable to reverse disease progression (39). IL-10 delivery can inhibit bleomycin-induced pulmonary fibrosis by suppressing the production of TGF-β1 from alveolar macrophages, lung fibroblasts, and myofibroblasts (40). Conversely, IL-10 deficiency accelerates the progression of bleomycin-induced pulmonary fibrosis in mice (41). The combination of IL-10 with rapamycin has been shown to inhibit the progression of pulmonary fibrosis with greater efficiency (42). These findings suggest that IL-10 has the potential to become a new antifibrotic agent.

According to conventional criteria for lung transplantation, donor lungs with high levels of pro-inflammatory cytokines are often excluded from transplantation (43), as transplanting such lungs significantly increases the 30-day mortality rate of recipients (44). In the context of donor lung shortages and the increasing maturity of ex vivo lung perfusion (EVLP) technology (45), researchers conducted EVLP using porcine donor lungs and administered adenovirus-mediated IL-10 (AdhIL-10) into the bronchi. This strategy significantly improved donor lung function, with no apparent adverse effects observed at the multi-organ level (46).

### 2.3 IL-10 formulations

Endogenously secreted IL-10 plays a crucial role in limiting excessive inflammatory responses, but its plasma concentration is influenced by multiple factors such as pathological states, its half-life, and signal regulation. Like many cytokines, IL-10 faces key limitations in therapeutic applications, including signaling redundancy, pleiotropic effects, and a narrow therapeutic window resulting from its short half-life (47). Therefore, relying solely on endogenous IL-10 is often insufficient to meet therapeutic needs, and exogenous IL-10 formulations are necessary for treatment.

#### 2.3.1 Recombinant human IL-10

Recombinant human IL-10 (rhIL-10) is produced in Escherichia coli that has been transfected with plasmids carrying the rhIL-10 gene. The produced IL-10 protein is identical to natural human IL-10, except for one methionine residue at the N-terminus. Recombinant mouse IL-10 (rmuIL-10) shares about 73% amino acid sequence homology with rhIL-10 (48). While rhIL-10 still retains some activity in mouse cells, rmuIL-10 does not exhibit significant effects in human cells (49). Therefore, rhIL-10 is the most commonly used form in current research. However, unmodified rhIL-10 has a terminal half-life of only 2.3 to 3.7 hours *in vivo* (13), which severely limits its sustained therapeutic effects in clinical practice. In a psoriasis treatment study, only high-dose and frequent administration regimens could maintain a relatively ideal therapeutic effect (50).

#### 2.3.2 IL-10 gene therapy strategies

IL-10 gene therapy strategies often use plasmids, lentiviruses, or adeno-associated viruses (AAV) as vectors to deliver the IL-10 encoding gene into host cells, with the primary goal of increasing endogenous IL-10 expression. Recombinant adenoviruses are typically constructed using standard homologous recombination methods (51). To generate recombinant adenoviral vectors expressing rhIL-10, a common approach is to isolate the cDNA sequence encoding IL-10 from the pDSRG-IL-10 plasmid (52). The safety of adenovirus gene therapy has been confirmed by several studies (46, 53). Lentiviruses, a type of retrovirus, can also be used to mediate IL-10 gene therapy (54). To construct a lentivirus that drives human IL-10 expression, IL-10 cDNA is amplified by PCR and inserted into the lentiviral backbone vector, followed by packaging using a classic three-plasmid system (55). The clinical safety of lentiviral gene therapy has also been established (56). Plasmids, which are double-stranded circular DNA molecules with autonomous replication capabilities, are commonly used as high-efficiency expression vectors for transient transfection (57) and to mediate IL-10 gene expression (58).

#### 2.3.3 IL-10 fusion proteins

**Figure 3:**
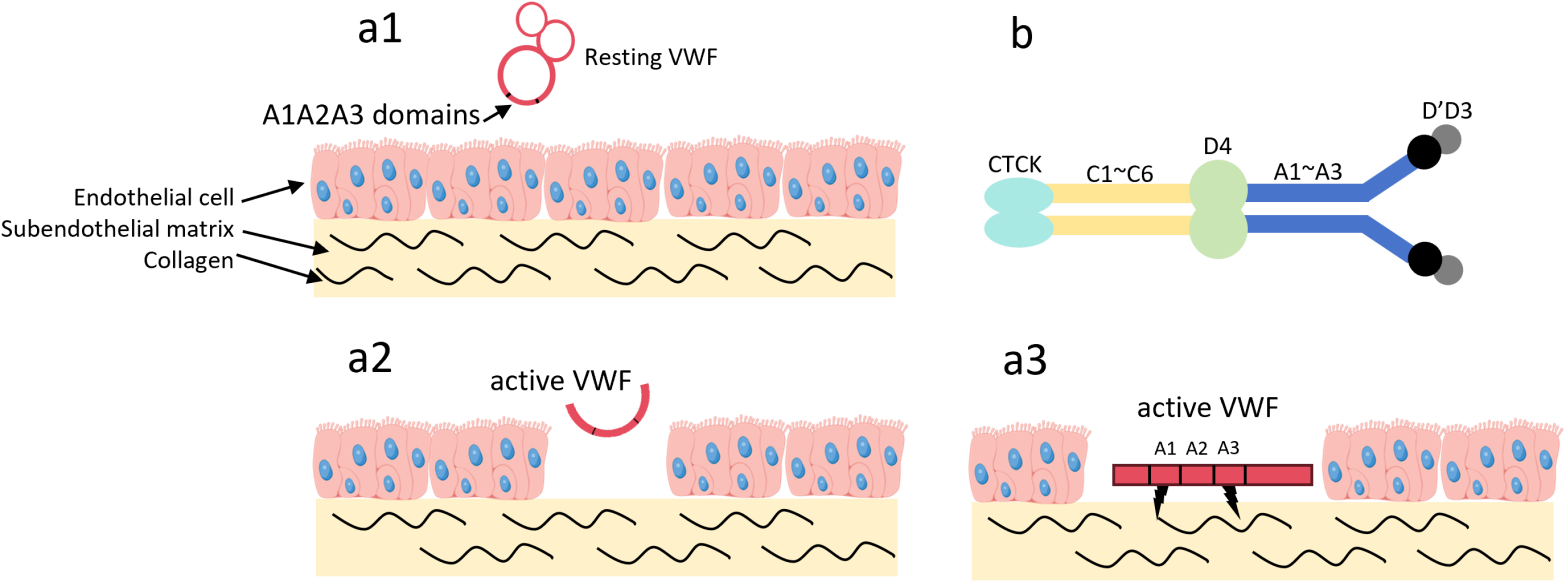
The VWF A3 domain can target type I and type III collagen. **(a1)** In the absence of vascular wall injury, VWF circulates in the blood in its resting state. In this state, VWF adopts a globular conformation, and the A1 and A3 domains are not exposed; **(a2)** Upon vascular wall injury, high shear stress activates VWF, and collagen in the subendothelial matrix becomes exposed; **(a3)** The A1 and A3 domains of VWF bind to the exposed collagen; **(b)** VWF exists as a dimer. Among these domains, the A1 domain can target type I, IV, VI collagen, while the A3 domain can target type I, III collagen. Von Willebrand Factor, VWF; C-terminal Cysteine Knot domain, CTCK.

These modified cytokines typically have a longer half-life and can enhance the efficacy of IL-10 in specific disease models. Vascular leakage in fibrotic tissue exposes extracellular matrix (ECM) proteins that are typically found in the blood circulation (59, 60), and von Willebrand factor A3 (VWF-A3) can bind to type I and type III collagen (61). **Figure 3** illustrates how the VWF-A3 domain enables VWF dimers to target collagen exposed at sites of vascular injury.

Michael J. V. White and his team (42) designed a recombinant protein by re-engineering the VWF-A3 domain and fusing it with IL-10. This A3–IL-10 fusion protein can target the leaky vasculature within fibrotic tissue. Using fluorescent labeling as a tracking method, the presence of VWF-A3 increased drug targeting to fibrotic lungs by approximately 3%, with a statistically significant difference compared to the non-fibrotic control group. Based on histological image analysis, the ratio of fibrotic to healthy tissue area in the A3–IL-10 treatment group improved by about 30% compared with the model group, the absolute collagen content was significantly reduced, and the Ashcroft score decreased by 2 points. The covalent attachment of polymers to proteins can enhance their bio-distribution when applied to tissues, and maintain their bioactivity over a longer period (62). Polyethylene glycol (PEG), formed by linking ethylene glycol molecules through ether bonds, is a neutral linear polyether with a broad range of molecular weights. The repeated ethylene groups in the PEG chain create hydrophobicity, while the oxygen atoms strongly interact with water (63). PEG conjugation prolongs the retention time of proteins in the lungs by promoting mucosal adhesion, increasing protein loading, and maximizing inhaled doses. It also reduces the uptake of PEG-conjugates by alveolar macrophages, significantly extending the protein’s half-life (64). High molecular weight PEG conjugated with drugs in a polymer-drug coupling can further extend the drug’s retention time in the lungs (65–67). PEG can be covalently linked to IL-10 to form PEGylated IL-10 (PEG-IL-10). PEG-IL-10 can mediate CD8+ T-cell cytotoxicity and promote IFN-γ expression in CD8+ T-cells, mediating tumor rejection and sustained tumor immunity (68). This mechanism shows promise for IL-10 in anti-inflammatory therapies as well. Immunoglobulin fragment crystallizable (Fc) fusion is another method to extend IL-10’s half-life. Mutations in the Fc domain can reduce antibody-dependent cellular cytotoxicity and complement-dependent cytotoxicity. By linking the C-terminus of the Fc domain to wild-type IL-10 via a glycine-serine-rich peptide linker, IL-10 activity can be preserved (69). The non-target-mediated clearance of Fc-rhIL-10 is estimated to be approximately 200 times slower than that of rhIL-10, leading to target-mediated drug disposition (TMDD) phenomena in the pharmacokinetics of the fusion protein (70). Current studies on the efficacy of IL-10 fusion proteins mainly focus on cancer (71), but their ability to mediate CD8+ T-cell proliferation, activation, and metabolic reprogramming (72) also gives IL-10 fusion proteins significant potential in the anti-inflammatory field.

## 3 IL-10 delivery systems

Based on past experiences with the failure of using rhIL-10 to treat diseases such as Crohn’s disease (73), rheumatoid arthritis (74), and sepsis (75), the systemic administration of IL-10 faces numerous challenges, necessitating the development of more effective delivery systems. Given the cytokine nature of IL-10, an ideal delivery system should be capable of extending its half-life, improving its bioavailability, and reducing the pleiotropic effects of the cytokine, thereby enhancing its anti-inflammatory effects and clinical application potential in the treatment of pulmonary diseases.

### 3.1 Nanoparticle delivery systems

Polymeric nanoparticles (PNPs), liposomes and lipid-based nanoparticles are widely used as typical nanocarrier systems (76). Liposomes are spherical vesicles composed of at least one layer of lipid bilayers. They vary in size from nanometers to micrometers and are primarily composed of phosphatidylcholine or lipids (77). Liposomes offer excellent biocompatibility, can encapsulate both hydrophilic and lipophilic agents, and exhibit minimal toxicity (78). In the treatment of pulmonary diseases, they can extend the local effect and reduce irritation to lung tissue (79). Inhalation administration is a more effective method for delivering liposomes to the lungs; however, intraperitoneal injection of liposomes carrying IL-10 gene expression plasmids can still alleviate lung injury in a mesenteric ischemia-induced ALI mouse model. In the study by Burhan Kabay et al. (80), mice injected with liposome-encapsulated hIL-10 plasmid DNA exhibited an increase in plasma hIL-10 concentration to 12.8 ± 1.28 ng/ml at the time of sacrifice. The lung wet-to-dry weight ratio in animals receiving the empty vector was significantly elevated to 7.47 ± 0.35, whereas the ratio in the treatment group was 4.26 ± 0.47. This indicates that the therapeutic intervention markedly improved pulmonary edema in ALI mouse. The activity of myeloperoxidase (MPO) was determined using a spectrophotometric method, and the treatment group exhibited approximately a twofold reduction in MPO activity compared with the empty vector group. The 2-hour survival rate in the treatment group was increased by 30% compared with the empty vector group. Lipid nanoparticles (LNs) are lipid-based nanocarriers. LNs are colloidal carriers made of biodegradable lipid matrices that are considered Generally Recognized as Safe (GRAS) and are stabilized by surfactants (81). LNs offer advantages such as high stability, large drug loading capacity, and non-toxicity (82), and although some LN-based platforms have been developed to enable targeted delivery to the lung (83–86), no studies have yet explored their use in IL-10 delivery or pulmonary disease treatment. Poly (D,L-lactic-co-glycolic acid, PLGA) copolymers emphasize biocompatibility and biodegradability (87), which can extend the *in situ* retention time of drugs and enhance mucosal penetration (88). IL-10 encapsulated in PLGA-chitosan can inhibit IL-6 and TNF-mediated inflammatory responses (89). Inhalation of PLGA-encapsulated MSC-derived exosomes has been shown to inhibit airway inflammatory responses (90). Poly-lactic acid-polyethylene glycol (PLA-PEG) is an amphiphilic polymer, and the use of PLA-PEG to encapsulate IL-10 in polymer nanoparticles significantly extends the half-life of IL-10 and improves its thermal stability (91). Micelles are relatively less common in IL-10 delivery; they are colloidal particles composed of amphiphilic block copolymers or surfactants (92), typically ranging in size from 5 nm to 100 nm. Micelles can prevent alveolar macrophage uptake and prolong drug release (93). In a mouse model of pulmonary fibrosis, intravenous injection of collagen-conjugated micelles carrying VWF-A3 fused IL-10 (A3-IL-10) significantly improved the degree of pulmonary fibrosis. **Table 1**. compares the advantages and disadvantages of the nanoparticles discussed in this section.

**Table 1:** Comparison of Different Nanoparticles.

Nanoparticle type	Advantages	Disadvantages/Challenges
Liposomes	Amphiphilic drug-loading capability Good incompatibility Low toxicity	Limited stability Easily cleared by reticuloendothelial system
Lipid Nanoparticles	High stability High drug loading Non-toxic	Higher cost preparation relies on precise microfluidic methods
Polymeric Nanoparticles	Excellent sustained-release properties Highly tunable material characteristics Good biocompatibility	Limited loading capacity for hydrophobic drugs Challenges in *in vivo* clearance Complex preparation
Micelles	Efficient loading of hydrophobic drugs Small size facilitates tissue penetration Simple preparation	Low stability Difficult to load hydrophilic drugs Relatively low drug-loading capacity

### 3.2 Gel delivery systems

Hydrogels are a class of non-Newtonian fluids composed of three-dimensional hydrophilic networks that store large amounts of water (94). With over 75% water content, hydrogels exhibit excellent biocompatibility (95) and are ideal materials for mimicking the extracellular matrix (ECM). In the context of chronic inflammation induced by surgical tissue damage, polyethylene glycol (PEG) hydrogel systems delivering IL-10 can promote the recruitment and functional re-education of monocytes in the damaged area, demonstrating potential for regulating regenerative cell subpopulations and promoting wound healing (96). Hyaluronic acid hydrogels can enhance the targeting and bioavailability of IL-10, demonstrating significant anti-inflammatory effects both locally and systemically (97, 98). In a bleomycin-induced mouse pulmonary fibrosis model (40), a hyaluronic acid–heparin hydrogel was used as a carrier for IL-10 (HH-10), and intranasal administration ensured its sustained release. IL-10 inhibited transforming growth factor-β (TGF-β)-driven collagen production in pulmonary fibroblasts and myofibroblasts, effectively reducing collagen deposition in the lungs and significantly improving both the degree of fibrosis and the 21-day survival rate in the treatment group. In the model group, approximately 1800 μg of collagen per gram of lung tissue was detected, whereas this value decreased to 1000 μg in the HH-10 treatment group. The Ashcroft score was reduced from 6 in the model group to 2 in the HH-10 treatment group. Self-assembling peptides (SAPs) represent another form of hydrogel, capable of spontaneously forming cross-linked nanofibers in aqueous solution, which then transition into three-dimensional hydrogels in physiological saline environments (99). IL-10 encapsulated in SAP hydrogels can also significantly reduce systemic pro-inflammatory cytokine levels (100). Similar to hydrogels, nanogels are submicron colloidal formulations obtained by micronizing the three-dimensional hydrogel network (94), and they can also be used as carriers for drug delivery (101). Research has shown that β-glucan nanogels carrying rhIL-10 can maintain a stable IL-10 concentration in mice for at least 4 hours (102), though no studies have yet explored the use of nanogel delivery systems for pulmonary anti-inflammatory therapy.

### 3.3 Extracellular vesicles and other delivery systems

Extracellular vesicles (EVs) are naturally derived carriers that play a key role in mediating intercellular communication, sharing molecular cues with their donor cells (103, 104). EVs can directly cross biological barriers to deliver various active biomolecules, with no significant size limitations (105). This allows EVs to overcome challenges faced by other delivery systems, such as nanoparticles. Mesenchymal stem cell (MSC)-derived EVs are the most common type, and even without carrying anti-inflammatory cytokines or therapeutic drugs, EVs and their derived nanovesicles (NVs) can suppress systemic inflammation (106, 107). MSC-EVs have demonstrated good efficacy in ALI (108, 109), lung transplantation (110), and asthma (111), with the anti-inflammatory effects being mediated by IL-10. When EVs are used as carriers for IL-10, they fully retain its immunomodulatory function (112), allowing IL-10 to penetrate biological barriers that it would not normally cross (113). Chitosan/alginate-modified EVs can protect IL-10 from premature degradation by the body’s environment (114). These findings make EVs direct as carriers for IL-10 in the treatment of pulmonary inflammation highly promising. In studies using a porcine lung transplantation model (115) and an ex vivo human lung model (116), Antti I. Nykänen and his team developed engineered mesenchymal stem cells capable of expressing IL-10, designated as MSC^IL-10^. This engineered MSC technology involves adenoviral transduction of human umbilical cord-derived mesenchymal stem cells (hUC-MSCs) to produce higher levels of human IL-10 (hIL-10). These cells maintained high levels of IL-10 secretion for at least six days even after cryopreservation and thawing, and are able to stably express IL-10 in ex vivo human lungs. When MSC^IL-10^ was used during EVLP, IL-10 levels in the perfusate increased significantly without negatively affecting lung function. In EVLP, a single administration of 20×10^6^ MSC^IL-10^ markedly reduced pulmonary apoptosis, whereas increasing the dose to 40 × 10^6^ promoted apoptosis. However, the therapeutic effect remained limited. This was mainly due to the difficulty in reversing elevated pro-inflammatory cytokine levels in poor-quality donor lungs, the detrimental impact of impaired donor lung metabolism on MSC^IL-10^ function, the challenge of maintaining IL-10 levels in a low-pH environment (pH < 6.4), and the inability of MSC^IL-10^ to survive for more than 3 days after administration via EVLP and subsequent lung transplantation. Overall, these studies open new avenues for IL-10-based cell delivery and the treatment of post–lung transplantation complications.

## 4 Targeting approaches

### 4.1 Nasal administration

Nasal administration, whether by nasal drops or aerosol inhalation, is the most direct targeting method. It offers advantages such as reducing drug dosage, improving patient compliance, and avoiding first-pass metabolism in the liver (117). This non-invasive route is particularly suitable for drug delivery platforms like polymer nanoparticles, liposome carriers (118), and gel carriers (40), which do not have intrinsic targeting functions. Although IL-10 is a pleiotropic cytokine, in an ALI mouse model, Huan Qin and colleagues (25) demonstrated that direct inhalation of exogenous IL-10 could markedly ameliorate pulmonary cytokine storm, lung edema, and histopathological damage. Two different doses of rhIL-10 (100 μg/kg and 200 μg/kg) were used for treatment, and the results showed that the high-dose group could reduce plasma levels of inflammatory cytokines to nearly those of the blank control group. Compared with the model group, in the high-dose treatment group, IL-1β decreased from 500 pg/mL to 100 pg/mL, IL-6 from 1200 pg/mL to 200 pg/mL, IL-8 from 800 pg/mL to 100 pg/mL, and TNFα from 1300 pg/mL to 100 pg/mL. The potential mechanism of this treatment is that rhIL-10 promotes the interaction between neutrophils and platelets through the STAT/SOCS–IκB/NFκB–CD40 signaling pathway, thereby facilitating the differentiation of neutrophils into an anti-inflammatory phenotype. While safety verification is still lacking for this approach, its simple administration method and efficacy make IL-10 aerosol inhalation a promising candidate for clinical translation. In a mouse model of asthma induced by ovalbumin sensitization (119), intratracheal administration of IL-10 significantly suppressed the infiltration of eosinophils and neutrophils as well as the development of airway hyperresponsiveness. This effect may occur through the suppression of proliferation in pulmonary vascular endothelial cells that express vascular cell adhesion molecule-1 (VCAM-1) and intercellular adhesion molecule-1 (ICAM-1). Although IL-10 did not improve airway remodeling in this model, it effectively attenuated airway hyperresponsiveness in corticosteroid-insensitive asthmatic mice. This alternative therapy may hold clinical potential for treating corticosteroid-insensitive asthma.

### 4.2 Immunogenic reutilization

**Figure 4:**
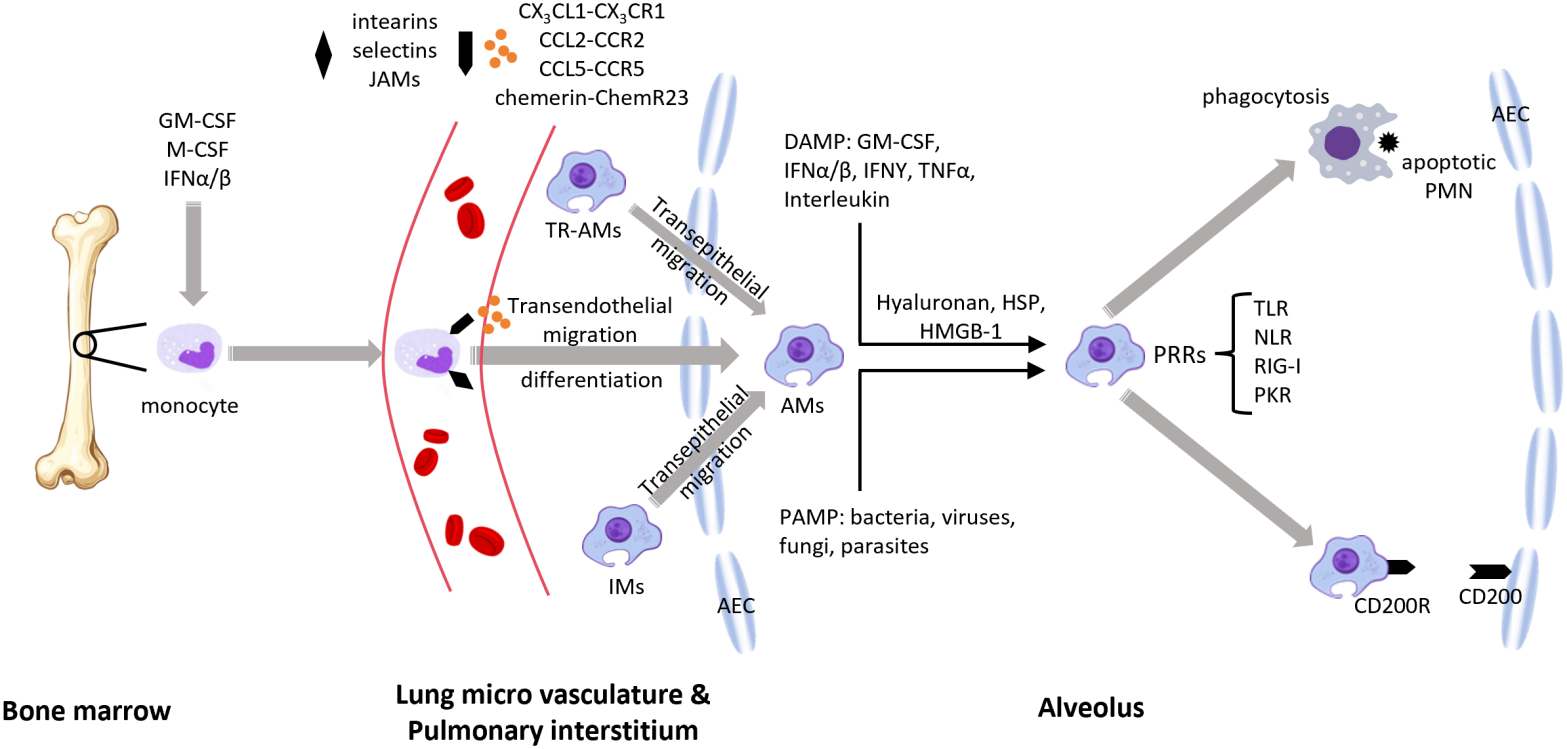
During pulmonary inflammation, lung macrophages from different origins can be recruited to the site of inflammation (1). Growth factors such as GM-CSF, M-CSF, and IFNs drive the differentiation and activation of macrophage progenitors (e.g., monocytes), which then enter the bloodstream (2). Macrophages are recruited to the alveoli under the influence of chemokines and their receptors, the ChemR23–chemerin axis, and adhesion molecules (3). Resident alveolar macrophages and interstitial macrophages are also recruited to the alveolar space (4). Pathogen-associated molecular patterns (PAMPs) and damage-associated molecular patterns (DAMP) transmit signals through (PRRs) (5). Macrophages phagocytose apoptotic neutrophils and promote an anti-inflammatory macrophage phenotype via the CD200–CD200R interaction. GM-CSF, granulocyte-macrophage colony-stimulating factor; M-CSF, macrophage colony-stimulating factor; IFN, interferon; JAMs, junctional adhesion molecules; TR-AMs, tissue-resident alveolar macrophages; AMs, alveolar macrophages; IMs, interstitial macrophages; DAMP, damage-associated molecular patterns; PAMP, pathogen-associated molecular patterns; HSP, heat shock proteins; HMGB-1, high-mobility group box 1; PRRs, pattern recognition receptors; TLR, toll-like receptor; NLR, NOD-like receptor; RIG-I, retinoic acid-inducible gene I; PKR, protein kinase R; PMN, polymorphonuclear neutrophils; AEC, alveolar epithelial cell.

Some immune cells or cell-derived materials naturally have the ability to target inflammatory regions or the lungs. Certain IL-10 delivery methods directly exploit this capability for targeted lung delivery. One such method is macrophage membrane coating, which targets pulmonary diseases. The innate immune system mediates the initial response to infection, with immune cells primarily composed of macrophages, which have a lifespan of several months, and neutrophils, which live for about 48 hours (120). Macrophages possess active targeting capabilities, high immunocompatibility, and long circulation times. They primarily recognize pathogens and respond to infection and injury through pathogen-associated molecular patterns (PAMPs) (121) and damage-associated molecular patterns (DAMPs) (122). Macrophage membranes express P-selectin glycoprotein ligand-1 (PSGL-1), L-selectin, lymphocyte function-associated antigen-1 (LFA-1), integrins, and very late antigen-4 (VLA-4), facilitating adhesion to inflammatory cells (123). These immune mechanisms make the macrophage membrane an ideal material for coating IL-10 and IL-10 carriers. In fact, macrophage membranes, as targeting materials, were initially used for targeting lung cancer rather than pulmonary inflammation (124). **Figure 4** illustrates the recruitment of pulmonary macrophages in response to pulmonary inflammation.

**Figure 5:**
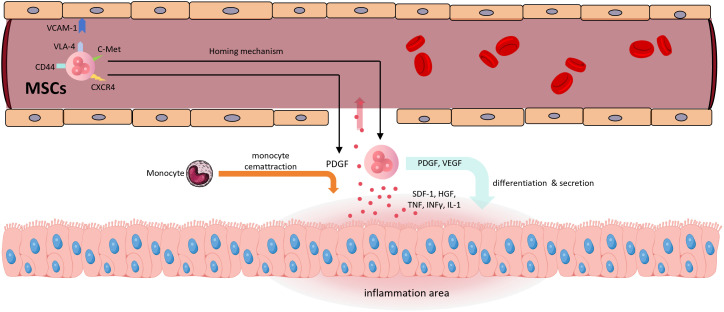
The homing mechanism of MSCs (1) Inflammatory regions release pro-inflammatory cytokines such as TNF, IFN-γ, and IL-1, as well as chemokines like SDF-1 and HGF, which enter the bloodstream (2). CXCR4 recognizes SDF-1, and c-Met recognizes HGF, initiating the homing mechanism (3). VCAM-1, VLA-4, and CD44 are involved in cell rolling, adhesion, and transendothelial migration (4). MSCs secrete PDGF and VEGF; the former promotes monocyte recruitment, while both contribute to the transition of inflamed tissue toward regeneration (5). MSCs themselves participate in tissue differentiation. SDF-1, stromal cell-derived factor 1; HGF, hepatocyte growth factor; CXCR4, C-X-C chemokine receptor type 4; c-Met, mesenchymal-epithelial transition factor; VLA-4, very late antigen-4.

However, Jun-Da Li and colleagues (125) demonstrated in a mouse model of house dust mite (HDM)-induced allergic airway inflammation that IL-10-loaded alveolar macrophage membrane-coated nanoparticles (IL-10-AMNPs) significantly reduced Th2 and Th17 cytokine levels, increased airway compliance and markedly reduced airway resistance, compared to IL-10-loaded poly(lactic-co-glycolic acid, PLGA) nanoparticles. IL-10-AMNPs reduced the total number of inflammatory cells in the bronchoalveolar lavage fluid by approximately 3.5-fold, from 3.5×10^6^ in the model group to 1×10^6^ in the treatment group. This therapeutic effect was significantly superior to that of the IL-10-PLGA nanoparticles (IL-10-NP) group without encapsulated pulmonary macrophage membranes. In terms of inflammatory factor improvement, IL-10-AMNPs showed significantly superior effects on IL-10, IL-13, IL-17A, TNFα, and IFNγ levels compared with both the model group and the IL-10-NP group. This confirmed that alveolar macrophage membranes could effectively target drug delivery to areas of pulmonary inflammation, significantly enhancing the overall therapeutic efficacy. Mesenchymal stem cell-derived exosomes (MSC-Exos) have been shown in numerous studies to be effective for treating pulmonary inflammation (126–128). MSCs can be derived from various cell types, and exosomes derived from adipose-derived mesenchymal stem cells (AdMSCs-Exos, 108) and bone marrow-derived mesenchymal stem cells (BMSCs-Exos, 129) naturally accumulate in the lungs, making them ideal natural targeted lung carriers. The mechanism behind the targeting ability of exosomes remains unclear, but it may be related to the homing effect of MSCs (130). MSC-EVs have been used in several studies for IL-10 delivery (112–114, 131), making exosome-mediated IL-10 delivery for pulmonary diseases a promising approach. **Figure 5** illustrates the homing mechanism of MSCs. MSC-derived exosomes are likely to inherit this homing mechanism to target sites of inflammation.

### 4.3 Engineered modifications

**Figure 6:**
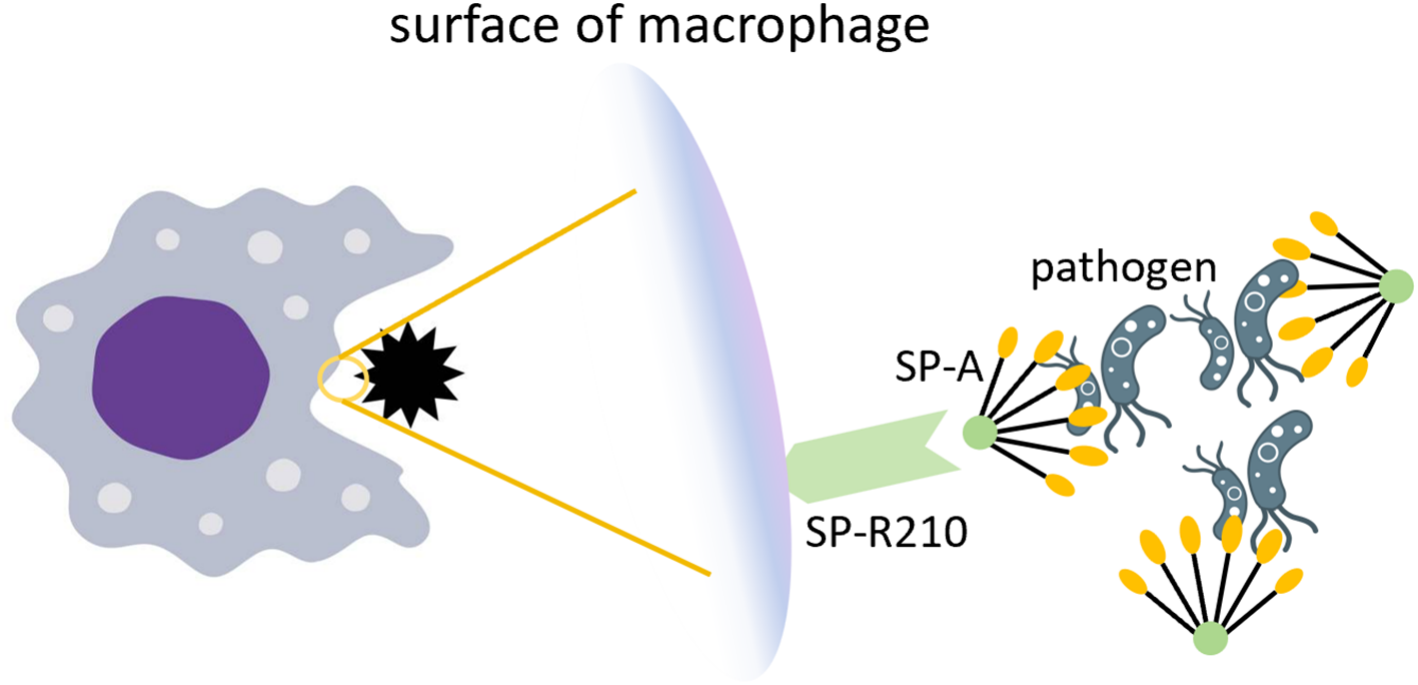
The role of SP-A in pathogen clearance. SP-A recognizes and aggregates pathogens by relying on the COOH-terminal carbohydrate recognition domain. The SP-R210 receptor on the surface of macrophages recognizes SP-A and enhances phagocytosis of pathogens.

The targeted engineering modifications of IL-10 carriers primarily depend on the specificity of the target organ and disease. In earlier animal experiments (132, 133), pulmonary surfactant protein A (SP-A) was confirmed to have targeting abilities for the lungs. SP-A helps maintain pulmonary homeostasis by acting as an innate immune scavenger receptor, regulating the expression of receptors, including the mannose receptor, on macrophages, which is a key receptor for mediating phagocytosis (134). SP-A can bind to the SP-R210 receptors on pulmonary macrophage, which are critical regulatory targets in pulmonary inflammation (135). **Figure 6** illustrates how SP-A facilitates pathogen clearance in the lung.

In fact, Ana I. Salazar-Puerta and her team (131) successfully electroporated a plasmid encoding SP-A into murine-derived skin fibroblasts (MDF), thereby generating EVs capable of targeting lung tissue. These MDF-derived engineered EVs (MDF-eEVs) delivered IL-10 via intranasal administration. The ability of SP-A-functionalized eEVs to preferentially accumulate in the lungs *in vivo* was evaluated by intranasal delivery of fluorescently labeled IL-10 + SP-A eEVs. Biodistribution analysis performed 12 hours post-delivery using an *in vivo* imaging system (IVIS) showed that, compared with non-functionalized eEVs, SP-A-functionalized eEVs exhibited significantly enhanced retention in the lungs. Compared with the model group, the treatment group showed significant improvement in the inflammatory factors interleukin-6 (IL-6) and tumor necrosis factor-α (TNFα), as well as in pulmonary inflammatory cell infiltration. Miji Kim and colleagues (136) combined SP-A with alveolar macrophages to develop a more powerful drug delivery carrier with enhanced targeting capabilities. **Table 2** summarizes IL-10-based therapeutic approaches and advanced delivery systems for pulmonary inflammatory disease.

**Table 2:** Overview of IL-10-Based Therapeutic Strategies and Delivery Systems for Pulmonary Diseases”.

Model	IL-10 delivery vehicle	Route of administration	Results	Ref.
Lung injury induced by intestinal ischemia-reperfusion in mice	Liposomes loaded with IL-10 expression plasmid	Intraperitoneal injection	The reduction of pulmonary edema, the decrease in pulmonary myeloperoxidase levels, and the alleviation of the severity of lung tissue damage pathology.	(80)
Bleomycin-induced pulmonary fibrosis in mice	VWF-A3-IL-10 fusion protein	Intravenous injection	The collagen content in the fibrotic lung lobes was significantly reduced, and the Ashcroft score was significantly decreased.	(42)
EVLP of human lungs	MSC^IL-10^	Pulmonary arterial injection	The treatment immediately increased the levels of IL-10 in the alveolar lavage fluid and lung tissue during EVLP, with no impact on lung function. However, the IL-10 levels were difficult to sustain in a low pH environment over time.	(116)
EVLP of pig lungs	MSC^IL-10^	Pulmonary arterial injection	The treatment rapidly increased hIL-10 during EVLP and caused a transient elevation of hIL-10 after lung transplantation, with no impact on lung function during the treatment period.	(115)
Bleomycin-induced pulmonary fibrosis in mice	Hyaluronic acid-heparin hydrogel loaded with rhIL-10	Intranasal administration	The survival rate at 21 days in the treatment group was significantly increased, the degree of pulmonary fibrosis was significantly reduced, and the total collagen content in the lungs was significantly decreased.	(40)
HDM inhalation-induced allergic airway disease in mice	Alveolar macrophage membrane-coated PLGA-IL-10 nanoparticles	Intranasal administration	The number of eosinophils and monocytes in BALF was reduced, and bronchial narrowing along with lung inflammation cell infiltration was significantly alleviated.	(125)
LPS-induced ALI	SPA-functionalized MDF-eEVs loaded with IL-10 expression plasmid	Intranasal administration	SPA functionalization significantly enhanced the pulmonary targeting and retention of eEVs. Tissue damage, pro-inflammatory cytokine secretion, macrophage activation, protein-rich fluid influx, and neutrophil infiltration into the alveolar space were significantly alleviated.	(131)
LPS-induced ALI	rhIL-10	Inhalation via nebulization	It significantly alleviated the cytokine storm in the lungs, reduced pulmonary edema, and mitigated the histopathological damage to lung tissue, potentially involving the regulation of the neutrophil STAT/SOCS-IκB/NFκB-CD40 signaling pathway.	(25)
asthma induced by ovalbumin sensitization in mice	rIL-10	Intranasal administration	The infiltration of eosinophils and neutrophils, as well as airway hyperresponsiveness, was significantly suppressed; it was effective in a corticosteroid-insensitive model but failed to improve airway remodeling.	(119)

## 5 Challenges in IL-10 delivery

### 5.1 Good manufacturing practice challenges of IL-10 vectors

MSC-based products, as living cells that undergo continuous changes over time, require more sophisticated risk management strategies to ensure product quality. Owing to the heterogeneity of MSCs themselves and their preparation methods, variations in extracellular vesicle (EV) production, and limited reproducibility of *in vitro* and *in vivo* functional assays, the efficacy and safety of MSCs remain highly controversial (137). As derivatives of MSCs, MSC-EVs are most commonly isolated and concentrated by differential ultracentrifugation. However, this method still faces several challenges, including low EV yield, reduced recovery following purification and washing, spontaneous aggregation of vesicles making resuspension difficult, structural disruption, and difficulty in scaling up production (138).

In the European Union, Regulation No. 1394/2007, introduced in December 2008, classified advanced therapy medicinal products (ATMPs), including MSCs, as medicinal products for regulatory oversight. Since 2018, guidelines for Good Manufacturing Practice (GMP) in ATMPs have been implemented (139). Recently, fully automated, closed Ficoll-based systems have been applied for harvesting bone marrow (BM) cells. Furthermore, a novel GMP-compliant non-woven filter system has shown promise in increasing cell yield without altering MSC characteristics (140). To meet large-scale expansion requirements, multilayered adherent culture systems that comply with GMP standards have been employed for MSC production (141). For MSC-EV production, size-based fractionation strategies, such as size exclusion chromatography and tangential flow filtration (TFF), are increasingly recognized as GMP-compatible and scalable technologies (142).

Lentiviral vectors play a crucial role in gene-modified cell therapies. Human embryonic kidney (HEK) 293 and its derivative 293T cell lines are the most widely used systems for lentivirus production. The first and still most commonly used lentiviral production system is based on transient production via multiplasmid cotransfection of 293T cells, which enables rapid and efficient vector generation (143). However, transient systems require large amounts of highly purified DNA and transfection reagents, making them unsuitable for large-scale, high-titer production. Stable producer cell lines (PCLs) remain the preferred choice for large-scale lentivirus manufacturing, though current limitations include batch-to-batch variability and elevated production costs (144).

Due to the clinical development of pegilodecakin and its completion of Phase III clinical trials, PEGylation of IL-10 no longer faces major GMP-related challenges (145). In contrast, PLGA-based systems present distinct obstacles. PLGA nanoparticles are primarily administered by injection (146). Given the physicochemical properties of PLGA, sterilization methods such as steam autoclaving or gamma irradiation often result in polymer degradation, making sterile filtration the optimal choice for PLGA nanoparticles (147). Moreover, as self-assembling drug delivery systems, PLGA nanoparticles require separation of free active pharmaceutical ingredients (APIs) from successfully assembled nanoparticles, for which diafiltration is the preferred method (148). This must be performed in conjunction with sterile filtration to comply with GMP standards.

### 5.2 Optimization of IL-10 therapeutic strategies

The therapeutic response to IL-10 is highly dependent on variations within the patient’s immune microenvironment, making individualized treatment a critical unmet need in clinical translation. The IL-10 gene comprises five exons (149). Numerous single-nucleotide polymorphisms (SNPs) and microsatellite polymorphisms have been reported in IL-10, including IL-10-1082G/A, IL-10-819C/T, IL-10-592C/A, IL-10.R, and IL-10.G (150). Interindividual variation in IL-10 levels is largely determined by SNPs within its promoter regionv (151). Thus, attention to IL-10 promoter and receptor SNPs provides direction for developing personalized therapeutic strategies.

Another major clinical translational challenge lies in IL-10’s pleiotropic effects on downstream signaling pathways. Formation of the IL-10/IL-10 receptor complex induces phosphorylation of STAT3 in macrophages, which can paradoxically activate pro-inflammatory cytokines such as IL-6 and prevent PI3K recruitment (47). IL-10 has also been implicated in the induction of human leukocyte antigen-G (HLA-G), an immune checkpoint molecule (152). HLA-G, by interacting with killer cell immunoglobulin-like receptors (KIRs), protects target cells from NK cell-mediated cytotoxicity (153), thereby attenuating innate immunity. Profiling cytokine networks downstream of IL-10 signaling may help clarify differences in patient immune environments.

Balancing IL-10’s anti-inflammatory efficacy with its potential immunosuppressive risks remains a critical challenge. c-MAF, a member of the MAF transcription factor family and part of the AP-1 superfamily, binds directly to the MAF recognition element (MARE) sequence within the IL-10 promoter in human macrophages (154). c-MAF regulates IL-10 expression directly and mediates the M2 macrophage polarization program induced by IL-10 (155). Interestingly, in cases of mild pulmonary inflammation (e.g., low-dose LPS), pulmonary macrophages exhibit low c-MAF expression, and IL-10 is not induced under these conditions (156). β-glucan curdlan, a c-MAF antagonist (157), has shown potential in mitigating immunosuppression arising from missed IL-10 therapeutic windows.

Moreover, engineered strategies provide novel approaches to optimizing IL-10 therapy. In one study, conventional CD4+ T cells were engineered with synthetic Notch (synNotch) regulatory circuits to respond to specific antigens (158). Upon antigen recognition, synNotch activation locally induced the production of customized anti-inflammatory payloads, including IL-10. These engineered T cells exhibited dual-antigen recognition of tumor cells: in the presence of Her2 alone, T cells exerted cytotoxic effects, whereas co-expression of Her2 and CD19 abrogated killing in favor of local immunomodulation. To effectively implement such promoter-driven localized expression systems, identification of unique antigenic epitopes in the target organ or tissue must precede payload selection.

### 5.3 Potential risks of the vector

Adeno-associated virus (AAV), a non-enveloped parvovirus, is currently one of the most widely studied gene delivery vectors. More than 255 clinical trials involving AAV-mediated gene therapy are ongoing, and seven AAV-based products have received regulatory approval (159). Lentiviruses, belonging to the Retroviridae family, are enveloped viruses with high transduction efficiency and the ability to confer stable, long-term transgene expression, making them advantageous in gene therapy. For instance, lentiviral delivery of chimeric antigen receptor (CAR) genes enables T cells to recognize and target tumor cells, and CAR-T therapies have been approved by the U.S. Food and Drug Administration (FDA, 160).

The primary challenges for viral vectors lie in the complexity of viral-receptor interactions, as the mechanisms by which enveloped viruses engage their receptors remain incompletely understood. Some viruses can bind multiple receptors or utilize alternative entry pathways, complicating engineering strategies. For AAV vectors, issues of delivery efficiency, packaging optimization, and host immune responses—particularly at high therapeutic doses—remain obstacles in clinical development (161).

Overall, hydrogel-based products hold great promise for advancing medical technologies, but further progress is required to facilitate translation from bench to bedside. Current challenges include material-associated immune-mediated foreign body responses (FBRs), leading to fibrosis around the hydrogel, impaired cellular infiltration, and ultimately therapeutic failure (162). Fortunately, modified alginate analogs demonstrate excellent biocompatibility, with negligible fibrosis observed (163).

## 6 Conclusions and future directions

Recent advances in IL-10 delivery strategies have significantly improved its therapeutic potential. Particularly promising are lung-targeted approaches, which enhance IL-10 accumulation in lung tissues while minimizing systemic exposure. Despite these advances, several hurdles remain. These include the need to optimize lung-specific targeting, extend the duration of IL-10 activity, ensure consistent therapeutic outcomes across disease models, and better understand potential long-term effects. Additionally, balancing IL-10’s immunosuppressive functions without compromising host defense or promoting tumorigenesis remains critical. Future study should prioritize: 1) Developing precision delivery systems: Next-generation delivery platforms should integrate prolonged bioactivity, biodegradability, and minimal immunogenicity to optimize IL-10’s therapeutic efficacy. LNs, polymeric nanoparticles (e.g., PLGA, PLA-PEG), and engineered EVs are promising candidates for delivering IL-10, as well as other anti-inflammatory, anti-fibrotic, or gene-editing agents, directly to lung tissue. 2) Personalized medicine approaches: Leveraging omics data (genomics, transcriptomics, proteomics) and artificial intelligence (AI)-based predictive models can enable tailored IL-10 therapies based on individual disease phenotypes and drug response profiles. By identifying biomarkers such as baseline IL-10 expression, immune cell signatures, or genetic variants, personalized dosing and delivery strategies can be developed to optimize efficacy and minimize adverse effects. AI-driven models could also predict patient-specific inflammatory dynamics, guiding the selection of IL-10 formulations or combination therapies for conditions like COPD or pulmonary fibrosis. 3) Targeting specific lung cell types: Precise targeting of IL-10 to specific lung cell populations - such as alveolar macrophages, epithelial cells, fibroblasts, or endothelial cells - can enhance therapeutic specificity and reduce systemic side effects. Emerging strategies include exploiting ligand-receptor interactions (e.g., SP-A binding to P63/CKAP4 receptors on type II alveolar cells) or cell-specific promoters to drive IL-10 expression in target cells. With continuous innovation in biomaterials and targeted delivery technologies, IL-10 is poised to become a key component of next-generation therapies for pulmonary inflammation. By addressing the remaining challenges, IL-10-based treatments have the potential to move from promising experimental strategies to effective clinical solutions for patients suffering from debilitating lung diseases.
